# Clinical Study of Serum Serotonin as a Screening Marker for Anxiety and Depression in Patients with Type 2 Diabetes

**DOI:** 10.3390/medicina58050652

**Published:** 2022-05-11

**Authors:** Lavinia-Alexandra Moroianu, Curis Cecilia, Valeriu Ardeleanu, Anca Pantea Stoian, Vasilica Cristescu, Raisa-Eloise Barbu, Marius Moroianu

**Affiliations:** 1 Hospital of Psychiatry “Elisabeta Doamna”, 290 Traian Street, 800179 Galați, Romania; 2 Clinical Medical Department, Faculty of Medicine and Pharmacy, University “Dunărea de Jos”, 47 Domnească Street, 800008 Galați, Romania; 3 Doctoral School, Faculty of Medecine, “Ovidius” University, 1 University Alley Street, Corp B, 900470 Constanta, Romania; valeriu.ardeleanu@gmail.com; 4 General Hospital “Căi Ferate”, 4-6 Alexandru Morutzi Street, 800223 Galați, Romania; 5 Arestetic Clinic, 78 Brailei Street, BR4A, 800108 Galati, Romania; 6 Diabetes, Nutrition and Metabolic Diseases Department, “Carol Davila” University of Medecine and Pharmacy, 8 Eroii Sanitari Street, 050474 Bucharest, Romania; ancastoian@yahoo.com; 7 School of Medicine and Pharmacy, University Titu Maiorescu, 22 Dambovnicului Tineretului Street, 040441 Bucharest, Romania; lali.cristescu@yahoo.com; 8 Pediatric Department, Emergency Clinical Pediatric Hospital “Sf. Ioan”, 2 Gheorghe Asachi Street, 800487 Galați, Romania; raisauibariu@gmail.com; 9 Department of Dental Medicine, Faculty of Medicine and Pharmacy, University “Dunărea de Jos”, 47 Domnească Street, 800008 Galați, Romania; moroianu.g.marius@gmail.com; 10 Medical Assistance Service of the Municipality of Galați, 97 Traian Street, 800112 Galați, Romania

**Keywords:** serotonin, type 2 diabetes, anxiety, depression, prognosis, comorbidities

## Abstract

Over time, studies have shown the importance of determining serotonin levels to diagnose somatic and psychiatric disorders. There are theoretical premises and practical ways to achieve a subtle correlation between the existence of comorbid psychiatric disorders and somatic diseases caused by the changes observed in serotonin levels. The present study, classified as retrospective and quantitative, provides evidence for determining the serotonin levels in patients with diabetes and anxiety or depression. A total of 48 patients with diabetes type 2 were enrolled in the study. Blood glucose level, glycated haemoglobin, and serum serotonin were noted, and they completed Hamilton A and Beck Depression Inventory questionnaires. We found robust correlations between serum serotonin and blood glucose (Sig. = 0.008), serum serotonin and HbA1c (Sig. = 0.007), serum serotonin and anxiety (Sig. = 0.000), and serum serotonin and depression (Sig. = 0.000). It is also noteworthy that women recorded extreme values higher than men for glycated haemoglobin (95% confidence interval: 6.92–7.79 in women and 6.30–7.23 in men). In conclusion, using serotonin as a marker of the mentioned diseases in clinical practice is of significant utility, considering the benefits in terms of the evolution and prognosis of comorbidities in patients with type 2 diabetes and anxiety and depressive symptoms.

## 1. Introduction

Serotonin (5-hydroxy-tryptamine) is a monoamine derived from the essential amino acid tryptophan, synthesised both in serotonergic neurons in the central nervous system and by enterochromaffin cells (Kulchitschy) distributed in the mucosa of the gastrointestinal tract. It is metabolised in the liver, and its main metabolite, 5-hydroxy indoleacetic acid, is excreted in the urine. Most of the serotonin in the blood is concentrated in platelets, from where it is released during the coagulation process. The main utility of determining serotonin in serum is diagnosing carcinoid syndrome. At the CNS level, it functions as a neurotransmitter, influencing mood and actively participating in monitoring the sleep–wake cycle [1,2]. Data from the literature indicate that serotonin levels under 100 μg/L can be associated with depressive syndromes. One in two studies found that elevated serotonin levels were correlated with a lower risk of suicide [3]. Psychopathology linked to physical conditions and the onset of chronic diseases is receiving growing attention since scientific evidence is confirming the need to consider psychic manifestations due to medical conditions as well as symptoms occurring due to the role of psychological domains and phenomena [4,5,6,7,8,9]. Serum serotonin values over 400 μg/L are usually associated with metastatic abdominal carcinoid tumours. Slight increases can be found in other clinical conditions such as intestinal occlusion, acute myocardial infarction, dumping syndrome, cystic fibrosis, and non-tropical sprue. Drugs that may affect serotonin levels are monoamine oxidase inhibitors, lithium preparations, methyldopa, morphine, and reserpine [2].

**Aim of the study:** This study aimed to determine if knowing the serotonin levels in patients with diabetes is useful as an advanced marker of diagnosis of depression and anxiety in clinical practice.

## 2. Materials and Methods

For all the 48 patients enrolled in this group, serotonin level tests were performed, the patients assuming the full costs of the analysis performed. Each of the subjects enrolled in the group was selected following the signing of the informed consent. The study was conducted in a public hospital with ambulatory service and a primary care unit.

In order to carry out the serum serotonin correctly, the patient must be prepared in advance. He will initially be asked to discontinue (if following such treatment) the administration of monoamine oxidase inhibitors (as they tend to increase serotonin levels) at least one week before harvest [1]. Serotonin analysis will be performed using venous blood (with a sample volume of at least 2 mL of serum), collected in a vacutainer without anticoagulant, which may or may not have a separating gel. After harvesting, the serum will be separated by centrifugation. Then the fresh serum will be worked (otherwise, it will be kept in special conditions at −20 °C or −70 °C, this being stable for 3 months in these conditions; however, thawing and refreezing are not permitted). The processing will be done by high-pressure liquid chromatography. The reference values used for the interpretation of the data in the studio are 90–240 μg/L [2].

This research is analytical and retrospective, over a period of 3 years, between 2015 and 2018. The study was conducted in the ambulatory service in a primary care unit, a public hospital. The patients diagnosed with diabetes were selected, aged between 18–45 years. For the collection and centralisation of data, observation sheets from the hospital archive were used. In order to create a centralised table with all the necessary data, personal follow-up sheets were prepared for each patient in which a series of characteristics of interest were noted for the concretisation of the study, among which we mention the following:

- Socio-demographic characteristics;
- Clinical data: completion of BECK depression inventory and Hamilton A questionnaire for anxiety;
- Metabolic parameters: HbA1c value, blood glucose value and Serum serotonin value.

To complete the Hamilton A questionnaire and Beck Depression Inventory, we conducted face to face interviews.

Criteria for inclusion in the study: age between 18–45 years, patients with diabetes type 2 diagnosed for more than one year, signing informed consent, completing the depression and anxiety questionnaires, performing serum serotonin on their payment regimen, patients following antihyperglycemic therapy (oral medication or/with insulin therapy).

Exclusion criteria from the study: under 18 years of age and over 45 years of age, patients with no discernment, undiagnosed patients with diabetes or patients with type 1 diabetes or maturity latent autoimmune diabetes (LADA), patients diagnosed with tumours of various types prior the study (especially digestive cancers that are serotonin secretory), complications of diabetes or other somatic diseases, patients who refused to execute serum serotonin. We have not excluded the patients diagnosed with cancer during the study because the type of their cancer was not a serotonin secretory type. Of our patients, two women had mammary cancer diagnosed with incipient forms and a man was diagnosed with melanoma.

## 3. Results

**Figure 1:**
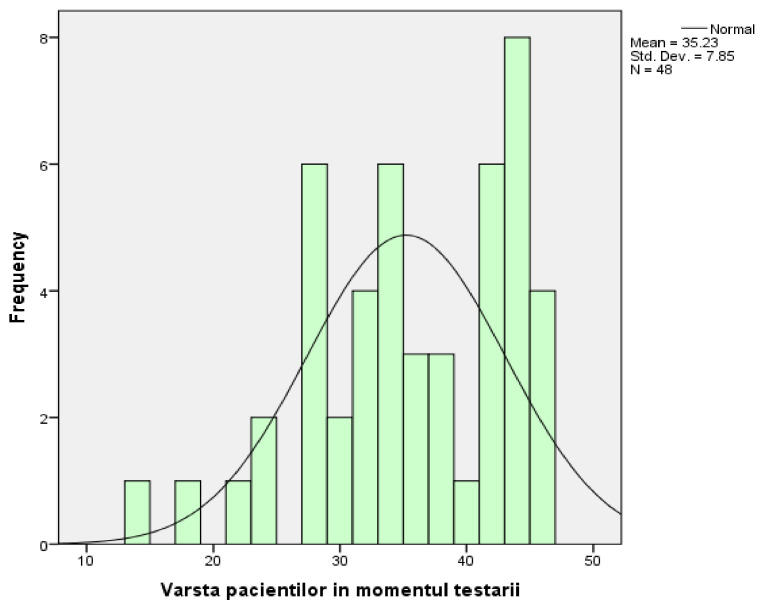
The frequency of patients’ declared ages at the time of testing.

The group in which the research took place consisted of 15 men (31.25%) and 33 women (68.75%). From the point of view of descriptive statistics, their age represents a variable characterised by the following parameters: the arithmetic mean of the ages is 35.23 with a standard deviation of 7.85 (Figure 1). The distribution curve for the study group is slightly asymmetrical to the left, which means that most people have ages grouped around the average, with a peak recorded on the right side of the distribution (the 8 people who are 44 years old).

Table 1 represents the general characteristics of the study.

In the next step, the incidence of the diagnosis of neoplasm as a pathology existing in the antecedents of the subjects was followed, and the information obtained reveals the following:

- Most of the patients studied (*n* = 39; 81.25%) did not have neoplasm;
- A total of 6 people (12.5%) show signs that make neoplasm a possible diagnosis;
- People (6.25%) were diagnosed with neoplasm during the study;
- By analysing the incidence of neoplasms depending on the sex of patients, the increased incidence (with an odds ratio of 2:1) in favour of the female sex was detected. Speaking of the vast majority of patients, meaning people who did not have cancer, 27 were women (representing a percentage of 81.82% of the total number of women), and 12 were men (80% of the total number of men). Analysing the percentages, one can observe an almost equal distribution of the sexes between the three categories:
  - 13.33% men compared to 12.12% women;
  - 6.67% compared to 6.06%;
  - 80% compared to 81.82%.

**Figure 2:**
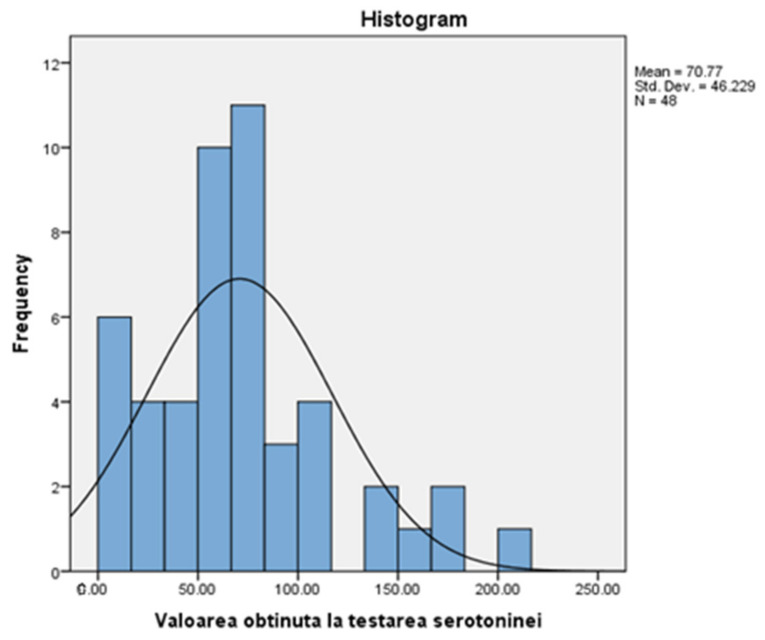
Values obtained when testing serotonin.

Table 2 presents a descriptive statistic of serotonin values, among which we mention: average (70.77), median (66.65), and standard deviation (46.22) for the 48 people in the group. Also, the adjacent figure (Figure 2) shows an asymmetrical distribution, inclined to the right, with most values placed below the normal level (90), specifically grouped around the mean (70.77). This means that most of the study group has a sub-standard level of serotonin.

**Figure 3:**
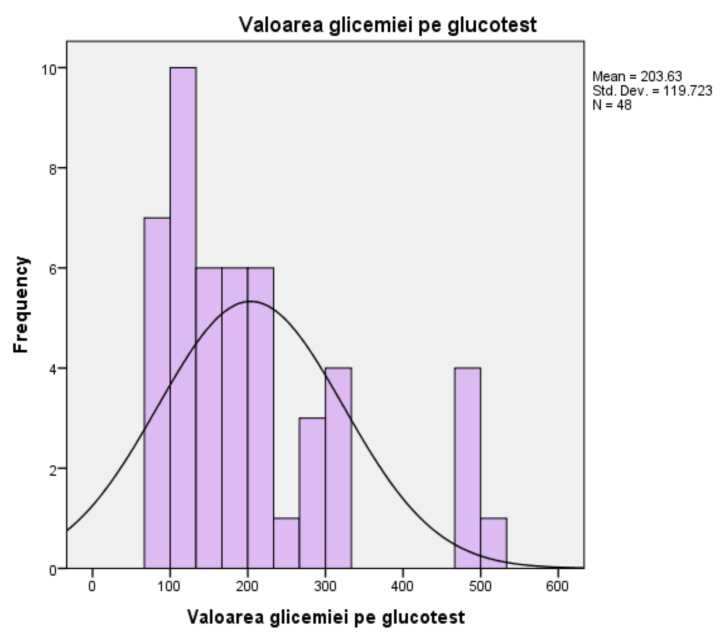
Blood glucose test results.

**Figure 4:**
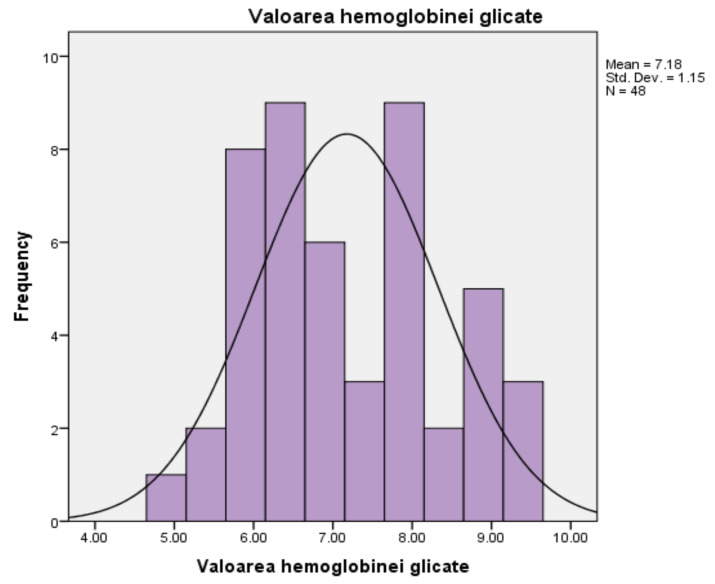
Results of HbA1c values.

- For blood glucose, measured in milligrams per deciliter, the mean score is 203.63 mL/dL, and the median is 169 mL/dL, with a standard deviation of ±119.72. The minimum value obtained in the glucose tests is 74 mL/dL, and the maximum value obtained is 501 mL/dL. The mean of 203.63 mL/dL shows an increased overall level, compared to the average level between 90 mL/dL and 126 mL/dL, for the tested population. The histogram follows a slightly asymmetrical curve to the right, meaning most scores are grouped to the left of the mean (203.63). Most people with the same value (10 people out of 48), representing the peak of the distribution curve, had blood glucose levels around 126 mL/dL, the upper limit of normal, the threshold indicating possible complications (Figure 3).
- For glycated haemoglobin, measured as a percentage, the persons in the studied group (*n* = 48) present an average of 7.17%, which exceeds the normality threshold, between 4% and 6.9%, respectively. The median is measured at a level of 6.95%, and the standard deviation is 1.149%. The minimum value recorded is 4.9%, while the maximum value recorded reaches the threshold of 9.2%. The corresponding histogram is characterised by the existence of a distribution curve that is slightly high and uniform, which means that the test results are evenly divided to the left and right of the mean (7.18%), and the extreme values are few in number (Figure 4).
- For glycated haemoglobin, measured as a percentage, the persons in the studied group (*n* = 48) present an average of 7.17%, an average that exceeds the normality threshold, between 4% and 6.9%, respectively. The median is measured at a level of 6.95%, and the standard deviation is 1.149%. The minimum value recorded is 4.9%, while the maximum value recorded reaches the threshold of 9.2%. The corresponding histogram is characterised by the existence of a distribution curve that is slightly high and uniform, which means that the test results are evenly divided to the left and right of the mean (7.18%), and the extreme values are few in number.

**Table 2:** Serotonin (μg/L) test values.

**N**	**Validity**	48
	**Lack**	0
**Average**		70.7729
**Median**		66.6500
**Standard deviation**		46.22853
**Minimum**		2.00
**Maximum**		209.00
**Sum**		3397.10

The statistical analysis presents the glucose and haemoglobin test results for patients included in the group (Table 3).

Above, by descriptive statistical analysis, we represent graphically (Table 4) the degree of anxiety according to the HAM-A scale, divided into four categories:

- Mild anxiety: 18 people (represents 37.5% of the study group);
- Average anxiety: 7 people (represents 14.6% of the study group);
- Severe anxiety: 15 people (represents 31.3% of the study group);
- Very severe anxiety: 8 people (represents 16.7% of the study group).

**Table 4:** Degree of anxiety according to the HAM-A scale.

		Frequency	Percentage	Valid Percentage	Cumulative Percentage
**Validity**	MA	18	37.5	37.5	37.5
	AA	7	14.6	14.6	52.1
	SA	15	31.3	31.3	83.3
	VSA	8	16.7	16.7	100.0
	**Total**	48	100.0	100.0	

Depression is another subject analysed in this group. In order to be able to visualise the incidence of this pathology by performing the BDI among the subjects, we have included Table 5, from which it can be observed:

- Mild depression: 18 people (represents 37.5% of the study group);
- Average depression: 7 people (represents 14.6% of the study group);
- Severe depression: 15 people (represents 31.3% of the study group);
- Very severe depression: 8 people (represents 16.7% of the study group).

**Table 5:** Degree of depression according to BDI.

		**Frequency**	**Percentage**	**Valid Percentage**	**Cumulative Percentage**
**Validity**	MD	10	20.8	20.8	20.8
	AD	14	29.2	29.2	50.0
	DS	14	29.2	29.2	79.2
	VSD	10	20.8	20.8	100.0
	**Total**	48	100.0	100.0	

For the categorical or ordinal type data, the Pearson correlation test (p) with a test for statistical significance at two extremes was applied under the previously known conditions (Table 6). There are statistically significant correlations for *p*-values of 0.01 between:

- The value obtained when testing serotonin with the value of glycemia on the glucose tests, the value of HbA1c, the degree of anxiety according to the HAM-A scale, the degree of depression according to the BDI;
- Blood glucose value on glucose test with the value obtained when testing serotonin, HbA1c value, degree of anxiety according to HAM-A room, degree of depression according to BDI;
- Value of HbA1c with the value obtained when testing serotonin, blood glucose value on glucose test, degree of anxiety according to HAM-A scale, degree of depression according to BDI;
- Degree of depression according to the BDI and degree of anxiety according to the HAM-A scale.

**Table 6:** Person type bivariate correlations.

		Sex	The Age of the Patients at the Time of Testing	The Value Obtained When Testing the Serotonin	Blood Glucose Value on Glucose Test	HbA1c Value	Degree of Anxiety Depending on the HAM-A Scale	Degree of Depression Depending on the BDI
**The value obtained when testing the serotonin**	Pearson Correlation	0.081	0.005	1	−0.377 **	−0.386 **	−0.612 **	−0.799 **
	Sig. (2 tailed)	0.583	0.973		0.008	0.007	0.000	0.000
	N	48	48	48	48	48	48	48
**Blood glucose value on glucose test**	Pearson Correlation	0.281	0.142	−0.377 **	1	0.815 **	0.457 **	0.465 **
	Sig. (2 tailed)	0.053	0.335	0.008		0.000	0.001	0.001
	N	48	48	48	48	48	48	48
**HbA1c value**	Pearson Correlation	0.238	0.255	−0.386 **	0.815 **	1	0.529 **	0.524 **
	Sig. (2 tailed)	0.103	0.080	0.007	0.000		0.000	0.000
	N	48	48	48	48	48	48	48
**Degree of anxiety depending on the HAM-A scale**	Pearson Correlation	0.082	−0.071	−0.612 **	0.457 **	0.529 **	1	0.734 **
	Sig. (2 tailed)	0.580	0.631	0.000	0.001	0.000		0.000
	N	48	48	48	48	48	48	48

** The correlation is significant at a yield of 0.01 (2-passes).

Table 7 shows the data obtained after performing the descriptive statistical analysis of the scalar variable defined by HbA1c, the sex of the patients enrolled in this study. Thus the following can be observed:

- On average, men (*n* = 15) have an HbA1c value of 6.77% (standard deviation of 0.84%), with a minimum recorded value of 5.4% and a maximum value of 8.2%;
- On average, women (*n* = 33) recorded HbA1c values of 7.35% (standard deviation of 1.23%), with a minimum documented value of 4.9% and a maximum of 9.20. It is noteworthy that women recorded extreme values higher than men.

**Table 7:** Glycated haemoglobin (%) value.

**Descriptive Values**								
**The Value of Glycated Haemoglobin**								
	**N**	**Average**	**Standard Deviation**	**Standard Error**	**95% Confidence Interval**		**Min**	**Max**
					**Upper Limit**	**Lower Limit**		
**Man**	15	6.7733	0.84216	0.21745	6.3070	7.2397	5.40	8.20
**Woman**	33	7.3576	1.23340	0.21471	6.9202	7.7949	4.90	9.20
**Total**	48	7.1750	1.14975	0.16595	6.8411	7.5089	4.90	9.20

The statistical value of the Levene coefficient (Table 8) is higher than the required threshold (of a Sig. of 0.01), which means that we can consult the result obtained after applying the ANOVA test (Table 9).

The ANOVA test for the value of HbA1c does not record a statistically significant result, so the null hypothesis is considered (Table 10).

## 4. Discussion

Regarding the structure data of the study, the group of patients consists of 48 adult patients: 15 men (31.25%) and 33 women (68.75%). Regarding the frequency of the declared ages of the patients at the time of testing, from the information existing after making the corresponding histograms, we can conclude that the arithmetic mean of the ages is 35.23 with a standard deviation of 7.85. The distribution curve is slightly asymmetrical to the left, which means that most people have ages grouped around the average, with a peak recorded on the right side of the distribution (the 8 people who are 44 years old).

Further, regarding the frequency of association of neoplasms as pathologies present concomitantly in the group of patients, the data provided by the analysis of the group show that the majority (*n* = 39; 81.25%) do not have neoplasm, 6 people (12.5%) have indices materialised in clinical signs that allow the establishment of neoplasia as a diagnosis, and 3 people (6.25%) are diagnosed with neoplasm. This type of pathology is suspected in two male and four female patients. Thus, similarly, in the case of gender distribution, female patients maintain a ratio of 2 to 1 compared to men. Concerning a particular type of neoplasm in the studied group in which there is a connection between diabetes—neoplasia—anxiety, and depression, we can refer to oesophagal neoplasm. It is well known that chronic alcohol consumption can cause complete glycoregulatory disorders with the appearance of diabetes and preneoplastic oesophagal lesions. In the present research context, the existence of both neoplasia and diabetes, per se, can be generators of anxiety disorders and depression [10]. In the present research context, analysing the percentages, one can observe an almost equal distribution of the sexes between the three categories: 13.33% men compared to 12.12% women; 6.67% compared to 6.06%; 80% compared to 81.82% compared to the 1997 Nishizawa Healthy Patient Study, which found that positron emission tomography showed that 52% higher serotonin synthesis in a healthy man than in a healthy woman, thus explaining the existence of a lower incidence of unipolar depression in men [11].

In addition, the histogram of the values obtained from the individual test of serotonin levels demonstrates the existence of an asymmetric distribution, inclined to the right, with most of the values placed below the average level (90), precisely grouped around the mean (70.77). This means that the majority of patients have a sub-standard level of serotonin, which plays an important role in the emotional state, as demonstrated both in the study and in the studies of other authors such as Dayan and Huys in 2008 [12]. A study conducted by professors at the University of Navarra, Pamplona (Spain) in 2018 on a sample of young obese patients showed that serotonin is an important factor in regulating body mass and glucose metabolism, so low serotonin levels lead to lower glucose, which is refuted in my research on diabetic patients because it has been shown that the higher the glycemic and HbA1c values are, the lower the serum serotonin level is [13].

Obesity and glucose metabolism are closely related to serotonin levels, an aspect highlighted in the present study. The literature provides evidence for the association of obesity with benign symmetrical lipomatosis caused by 90% of chronic alcohol consumption. Trauma caused at the psychological level by the association of obesity involved in the onset of diabetes, with benign symmetrical lipomatosis generated by alcohol consumption, can cause emotional disorders such as anxiety and depression through the phenotypic aspect that produces social isolation and impaired self-esteem, doubled by a biological picture that triggers symptomatology at the somatic level [14].

For patients enrolled in the study, in terms of blood glucose values, measured in milligrams per deciliter, the average score is 203.63 mg/dL, the median is 169 mg/dL, with a standard deviation of 119.72. The minimum value obtained with the glucose test is 74 mg/dL, and the maximum value obtained is 501 mg/dL. The average of 203.63 mg/dL shows an increased overall level, compared to the normal one between 90 mg/dL and 126 mg/dL, for the tested population. In this case, the distribution of the values recorded in the test using the glucose tests, which follows a slightly asymmetric curve to the right, means that most scores are grouped to the left of the mean (203.63). Most people with the same value (10 people out of 48), representing the peak of the distribution curve, had blood glucose levels around 126 mg/dL, the upper limit of normal, the threshold indicating possible complications. Distal symmetrical polyneuropathy is one of the most common complications of diabetes. Although it is relatively easy to diagnose, from the perspective of psychological implications, it is a significant reason for the occurrence of anxiety and depression by profoundly affecting the quality of life of this category of patients [15]. Data on HbA1c, measured as a percentage, show that patients (*n* = 48) have an average of 7.17%, which is above the normal threshold of between 4% and 6.9%, respectively. The median is measured at a level of 6.95%, and the standard deviation is 1.149%. The minimum value recorded is 4.9%, while the maximum value recorded reaches the threshold of 9.2%. Regarding the distribution of the recorded values of HbA1c for the subjects under study (*n* = 48), the distribution curve is slightly high and uniform, which means that the test results are evenly divided to the left and right of the mean (7.18%), and the extreme values are few in number.

The degrees of anxiety detected after completing the HAM-A scale are mild anxiety, with the highest percentage (37.5%), followed by severe anxiety (31.3%), very severe anxiety (16.7%), and average anxiety (14.6%). Regarding the degree of depression, quantified using the BDI, there is also a division according to the 4 categories: mild depression and moderate depression were assessed in the same percentage (29.2%) as in the case of severe depression and non-depressed patients (20.8%). A study published in the literature in early 2019 by Khan, Lutale, and Moledina on a group of 353 patients with diabetes found that the threshold for severe depression for their patients was not reached, which in the present study is well represented even with a proportion of 20.8% of patients who completed BDI and were diagnosed with this type of pathology [16].

Corroborating all these aspects, we can conclude that serotonin has an important predictive value in terms of influence on variations in HbA1c detected in patients in the group and glycemic values. Also, as can be seen from this table, there are signs of powerful statistically significant correlations (for *p* index values of 0.01) between the results obtained from serotonin tests, respectively, the values calculated for the BDI and HAM-A scale. From this point of view, we can adopt the hypothesis according to which, in the case of our group of patients, serotonin is an important marker in terms of emitting an alarm signal regarding a subsequent unfavourable evolution of the patients previously diagnosed with type 1 or 2 diabetes, an evolution that may involve the acquisition of psychiatric pathologies, such as depression or anxiety, quantified using scales completed individually. Furthermore, according to a study published by Tyano and collaborators in 2006, plasma serotonin levels combined with some psychometric scales (in the said study applying the BDI) can serve as a safe and inexpensive peripheral marker of psychopathology, as demonstrated in our study [17].

From a statistical point of view, there are significant correlations between serum serotonin and blood glucose, serum serotonin and HbA1c, serum serotonin and anxiety, and serum serotonin and depression. According to the literature, a study by Cowen P.J. clarified that low serum serotonin levels cause clinical symptoms of depression and that sometimes despite drug treatment, patients may experience relapses [18]. In the Iranian study conducted by Rezvanfar in 2010, the Hamilton D depression questionnaire was used in patients with type 2 diabetes, finding no valid correlation between depressive symptoms and HbA1c value [19]. The 2011 study by Yatan Pal Singh Balhara shows that the biggest challenge in managing psychiatric disorders among people with diabetes is the low detection rate, and up to 45% of cases of mental disorder and severe psychological distress are undetected among patients treated for diabetes. The prevalence of anxiety disorders in patients with diabetes is considerably higher than in the general population, and anxiety symptoms are significant risk factors for the development of diabetes [20]. Negative correlations were observed between the prevalence of anxiety disorders and HbA1c levels. Depression, anxiety and diabetes have a two-way causal association. Depression has been postulated to play a causal role in the onset of diabetes. The study shows that a meta-analysis reported that depressed individuals have an increased risk of a 60% increase in diabetes [21].

From the point of view of HbA1c values, men (*n* = 15) have, on average, a value of glycated haemoglobin of 6.77% (standard deviation 0.84%), with a minimum recorded value of 5.4% and a maximum value of 8.2%, and women (*n* = 33), on average, recorded glycated haemoglobin values of 7.35% (standard deviation 1.23%), with a minimum documented value of 4.9%, and a maximum of 9.20. It is noteworthy that women recorded higher values than men, a fact confirmed in the literature by the PREDATORR study in 2016, which determined a 17% higher share of diabetes in women than in men [22].

**Limits of the study:** The present study is not without limitations that may affect research and statistical analysis. First of all, our study did not have a controlled study. It should also be mentioned that the study is limited strictly to 48 patients with type 2 diabetes who assumed the full costs of the analysis performed, the reason for which the issuance of some conclusions is based on hypotheses detected strictly in the case of these patients. For this reason, from a future research perspective, we can consider the continuation of the study over an extended period of time and an increased number of subjects.

## 5. Conclusions

The present study provides evidence in support of data provided by previous studies regarding the benefits of using plasma serotonin levels as a diagnostic marker in correlation with psychometric scales for the diagnosis of psychopathological disorders. Another important aspect brought into discussion is the possibility of following the dynamics of evolution through clinical–biological–psychometric correlations and establishing the prognosis and intervention methods assessing the possible relapses of comorbidity type 2 diabetes—anxiety disorder and depression.

## Figures and Tables

**Table 1:** General characteristics of the study.

Gender (Male/Female)	15/33
Age (Year)	35.23 ± 7.85
Serum Serotonin value (μg/L)	70.77 ± 46.229
Blood glucose value (mg/dL)	203.63 ± 119.723
HbA1c value (%)	7.18 ± 1.15
Degree of anxiety depending on the HAM-A scale	18 (MA) 7 (AA) 15 (SA) 8 (VSA)
Degree of depression depending on the BDI	10 (MD) 14 (AD) 14 (DS) 10 (VSD)

Mean ± Standard Deviation, MA = Mild anxiety, AA = Average anxiety, SA = Severe anxiety, VSA = Very severe anxiety, MA = Mild depression, AD = Average depression, DS = Severe depression, VSD = Very severe depression.

**Table 3:** Results of glucose tests (mg/dL) and glycated haemoglobin (%) test for personnel in the analysed group.

		**Blood Glucose Value on Glucose Testes**	**The Value of Glycated Haemoglobin**
**N**	**Validity**	48	48
	**Lack**	0	0
**Mean**		203.63	7.1750
**Median**		169.00	6.9500
**Standard deviation**		119.723	1.14975
**Minimum**		74	4.90
**Maximum**		501	9.20
**Sum**		9774	344.40

**Table 8:** Variance homogeneity test.

**Variance Homogeneity Test**			
**The Value of Glycated Haemoglobin**			
**Levene Statistic**	**df1**	**df2**	**Sig.**
5.707	1	46	0.021

**Table 9:** ANOVA—The value of HbA1c.

**ANOVA**	**The Sum of the Squares**	**Df**	**The Average of the Squares**	**F**	**Sig.**
**Between groups**	3.520	1	3.520	2.763	0.103
**In groups**	58.610	46	1.274		
**Total**	62.130	47			

**Table 10:** Robust tests of equality of means.

**The Value of Glycated Haemoglobin**				
	**Statistical**	**df1**	**df2**	**Sig.**
**Welch**	3.655	1	38.568	0.063
**Brown-Forsythe**	3.655	1	38.568	0.063
a. Asymptotically F distributed.				

## Data Availability

All data presented in this study are available upon request.

## Abbreviations

HbA1c	glycosylated haemoglobin
CNS	central nervous system
BDI	Beck Depression Inventory
HAM-A	Hamilton A scale for anxiety
MA	Mild anxiety
AA	Average anxiety
SA	Severe anxiety
VSA	Very severe anxiety
MA	Mild depression
AD	Average depression
DS	Severe depression
VSD	Very severe depression
